# Supplementary material for: Discriminating three motor imagery states of the same joint for brain-computer interface
Source: PeerJ. 2021 Aug 24;9:e12027. doi: 10.7717/peerj.12027 (PMC8395581; doi:10.7717/peerj.12027)
Supplement: Supplemental Information 2 [file peerj-09-12027-s002.pdf]

Supplement 1 for Discriminating three imagery states of the same joint for brain-computer interface

Shan Guan, Jixian Li, Fuwang Wang, Zhen Yuan, Xiaogang Kang, Bin Lu

School of Mechanical Engineering, Northeast Electric Power University, Jilin City, Jilin Province, China

In this supplementary text, we present the examples of local mean decomposition (LMD) and selection of product functions (PFs).

1 Local Mean Decomposition

Taking the 3.0-6.0s data of FC6 electrode in experimental paradigm, which was induced by subjects' 7 shoulder abduction, as an example, LMD was employed to decompose the preprocessed EEG signal, and the extended part (the first n sampling points and the last n sampling points) was cut off to obtain the final PFs. The results are shown in Fig. 1A. Figure. 1B is the corresponding spectrum after FFT.

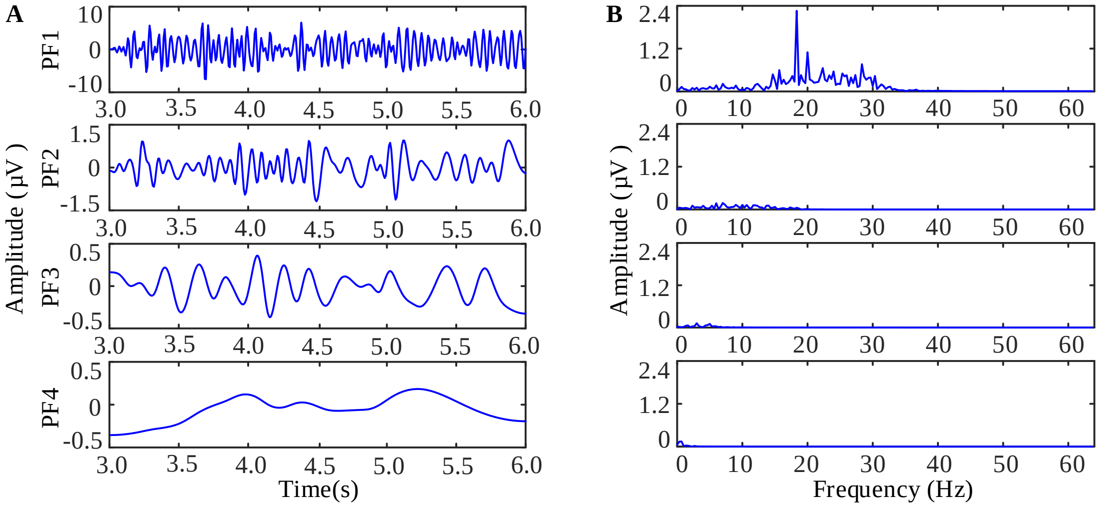

Figure 1 The first four PF components (A) and the corresponding spectrum (B). (A) The final product function (PF) components obtained from 3s EEG data after local mean decomposition (LMD). (B) The Corresponding spectrum of per PF of Fig. 1 (A).

2 Selection of Product Functions Based on the Cloud Model

Due to the inconsistent number of obtained PFs, we only calculated the cloud model parameters (Ex, En, He) of the first four PFs. Expectation (Ex) is the central value of a concept, entropy (En) represents the randomness of a qualitative concept, and super entropy (He) is the dispersion degree of a concept. Figure 2 shows the cloud model parameters of each PF component decomposed from the shoulder flexion MI data of subject 7. It can be seen from Fig. 2 (A) that the Ex of each PF component is similar and difficult to distinguish. However, it's obvious that the En and He of PF1 are much higher than the other PFs from Fig. 2 (B) and Fig. 2 (C). By analyzing a large amount of experimental data, we found that the results obtained are similar to the above results. Consequently, we selected the PF1 component as the effective

component for subsequent processing. At the same time, it can be found that α rhythm (8-12Hz) and β rhythm (13-30Hz) related to motor imagery are mainly distributed in PF1 by observing the spectrum of each PFs in Fig.1 (B). It can prove that the cloud model can effectively select the real PF component.

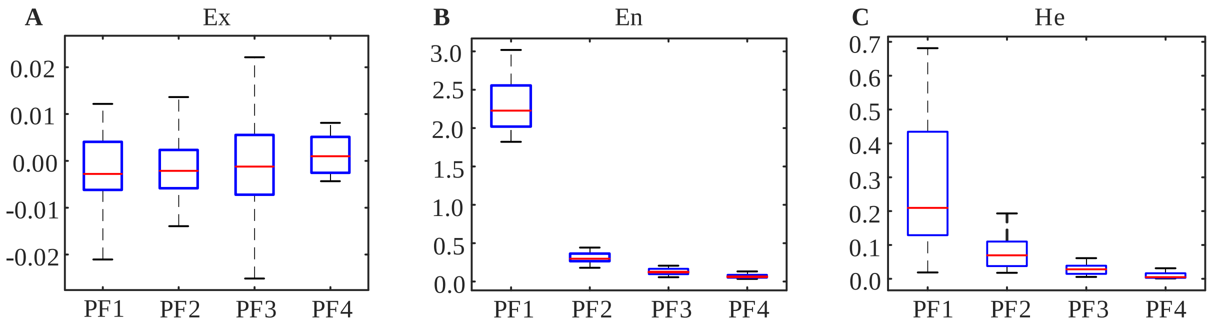

Figure 2 The box plot of the cloud parameters (Ex, En, He) of the first four product function (PF) component. (A) Expectation (Ex). (B) Entropy (En); (C) Super entropy (He).
